# Ovarian cancer mouse models: a summary of current models and their limitations

**DOI:** 10.1186/1757-2215-2-12

**Published:** 2009-09-28

**Authors:** Miranda Y Fong, Sham S Kakar

**Affiliations:** 1 Department of Physiology and Biophysics, James Graham Brown Cancer Center, University of Louisville, Louisville, KY 40202, USA

## Abstract

Development of mouse models representing human spontaneous ovarian cancer has been hampered by the lack of understanding of the etiology of this very complex disease. Mouse models representing the different types of ovarian cancer are needed to understand how epithelial ovarian cancer differs from granulosa cell tumors. Many different methods have been used to generate a viable genetic model with limited success. This review focuses on the methods of various investigators and the limitations of each model in establishing a reproducible and inheritable line to study this disease.

## Introduction

Ovarian cancer (OC) is the most lethal malignancy of the female reproductive system and the fifth leading cause of cancer death in women [1]. Ninety percent of OC are thought to arise from the epithelium and its inclusion cysts [2] due to multiple genetic changes [3]. However, the etiology of spontaneous epithelial (E)OC is poorly understood, partially due to a lack of an appropriate experimental model. While many approaches have been used, model development has been hampered by the absence of a specific promoter for the ovaries, as many promoters are sufficiently leaky. Numerous investigators have sought to develop a model that would effectively represent spontaneous human EOC. This review focuses on the methods various investigators have employed and the limitations of each murine model in establishing a reproducible, inheritable line to study this disease.

### Carcinogen induced tumor models

As early as 1969, ovarian tumors were induced by direct application of chemical carcinogens [4]. While 7,12-Dimethylbenz(a)anthracene (DMBA) had been used in 1970 to induce tumorigenesis in guinea pigs [5], a DMBA-coated suture was used in 1984 to induce ovarian tumorigenesis with only one of thirty-five mice developing an epithelial carcinoma [6]. However, despite these discouraging results, Nishita et al. [7] replicated this experiment by directly applying DMBA to the rat ovary using a coated suture. Nearly fifty percent of the rats developed ovarian tumors in 36 weeks, most of which were carcinomas. Unfortunately, DMBA also stimulated the epithelial surface of the fallopian tube, endometrium, and cervix to induce neoplastic transformation.

Other chemical carcinogens used to induce ovarian tumorigenesis include 20-methylcholanthrene, 1,3-butadiene, formic acid 2- [4-(5-nitro-2-furyl)-2-thiazolyl]hydrazide, a nitrofuran antibiotic, and N-methyl-N'-nitrosourea, a direct-acting alkylating agent [8-10]. To date, chemical carcinogens have not been associated with OC etiology [11].

### Syngeneic ovarian epithelial tumor models

Syngeneic models combine *in vitro *and *in vivo *methods to generate a tumor model. Briefly, mouse ovarian surface epithelial (MOSE) cells are isolated from the ovaries of virgin wildtype mice and cultured *in vitro *before transplantation into recipient mice [12]. Development of the mouse model was predicated on the work by Godwin et al. [13] and Testa et al. [14] on the spontaneous transformation of surface epithelial cells isolated from rats.

Roby et al. [12] established the technique of isolating and culturing MOSE cells, showing that MOSE cells can spontaneously transform *in vitro *with repeated passages and have tumorigenic capacity as they formed tumors and hemorrhagic ascitic fluid upon injection into athymic and C57Bl/6 receipt mice. This technique has been used by numerous investigators for subsequent studies [15-20].

Perhaps one of the most revealing MOSE studies was conducted by Roberts et al. [15], who compared the alterations of the actin cytoskeleton as well as expression of cellular adhesion proteins versus the number of passages to study the progression of ovarian carcinogenesis, showing that MOSE cells spontaneously transform with repeated passages. Late passage cells injected intraperitoneally into immunocompetent C57BL6 mice formed tumors in numerous organs, showing the transformation from a premalignant to a highly malignant phenotype with downregulation of E-cadherin and connexin-43.

Greenaway et al. [16] injected a spontaneously tumorigenic MOSE cell line, ID8, into the ovarian bursal cavity of C57Bl6 mice. The ID8 cells formed direct contact with the ovarian stroma, resulting in primary tumor formation, secondary peritoneal carcinomatosis, and extensive ascites fluid production between 80 to 90 days post-exposure. The cytological and architectural features resembled serous carcinoma. Interaction between ID8 cells and the ovarian stroma resulted in increased expression of proliferative and survival markers, including phosphorylated Akt, proliferating cell nuclear antigen (PCNA), and Bcl-2. Vascular endothelial growth factor (VEGF) levels were also increased in the serum and ascitic fluid. In conjunction, the pro-apoptotic factor Bax was decreased. The study supports the theory that the ovarian surface epithelium (OSE) can undergo invaginations and form inclusion cysts capable of undergoing neoplastic transformation [21].

### Genetically induced ovarian epithelial tumor models

One of the first reports to test genetic changes was made by Orsulic et al. [22], who used an avian retroviral delivery system. Transgenic mice were established to express the TVA virus receptor making them susceptible to infection to a subgroup of replication-competent avian leukosis viral-derived vectors (RCAS), thus allowing for the introduction of oncogenes that would integrate newly reverse-transcribed DNA into the host genome and allow long-term expression. The TVA receptor was placed under control of the keratin 5 promoter to direct expression to the ovarian epithelium or under control of the β-actin promoter to direct expression to all cells of the ovary. TVA-transgenic mice were crossbred with p53^-/-^mice to generate TVA/p53^-/-^, which were used to study the oncogenes *c-myc, K-ras*, and *Akt *individually and in combination. However, the keratin 5 promoter is constitutively active in the basal layer of stratified and simple epithelia in several organs [23]; therefore, it was necessary to isolate the expression of the virally delivered oncogenes. The ovaries were removed from the TVA/p53^-/- ^mice, cultured, and infected *in vitro *before introduction into recipient mice either by subcutaneous or intraperitoneal injection or by transplantation under the ovarian bursa. Once infected, the mammalian cells would not produce detectable levels of infectious viral particles, which limited spreading to the surrounding tissue. Introduction of any two oncogenes in keratin 5-TVA/p53^-/- ^ovarian cells was sufficient to drive tumorigenesis. While providing valuable insight into the genetics of tumorigenesis, this methodology is cumbersome at best. Because transplantation and *in vitro *manipulation are required, it is not possible to generate a stable transgenic line with an inheritable form of EOC.

Connolly et al. [24] used a novel approach to target simian virus 40 T antigen (SV40 TAg) to the epithelial ovarian surface by using the Mullerian Inhibitory Substance Type II Receptor (MISIIR) promoter. The Mullerian duct in the 8-week old embryo gives rise to female reproductive organs, including the fallopian tubes, uterus, and upper vagina. By linking the MISIIR promoter to the SV40 TAg, they were able to target expression of SV40 TAg to the epithelium of the female reproductive tract by microinjection of this construct into the male pronucleus of 0.5-day old embryos to generate transgenic animals. While 18 of 36 (50%) transgenic mice developed bilateral ovarian tumors resembling serous carcinomas by 6 to 13 weeks of age, the aggressiveness of this formation inhibited reproduction, making it extremely difficult to establish a transgenic line via the female founders. Two individual transgenic mice also developed a uterine mass and enlarged polycystic kidneys, respectively, possibly due to recombination events during transgenic mouse production. Not unexpectedly, 7 of 25 (28%) transgenic animals developed testicular cancer. Intrapleural invasion of tumors into the omentum, the mesentery, and visceral and parietal pleura was also observed, possibly due to the invasiveness of the ovarian tumors. However, SV40 TAg is not known to be a genetic contributor to ovarian carcinogenesis [3,25,26]. Yet despite these limitations, this model has been used for further experiments by establishing a transgenic line through the male founder [27,28].

Models that require either *ex vivo *manipulation or expression of a transgene during embryonic development do not accurately represent human EOC, which tends to be spontaneous in post-menopausal women. In an effort to mimic spontaneous EOC development, Flesken-Nikitin et al. [29] obtained mice from Anton Berns [30,31] with LoxP sites containing p53 and Rb alleles to assess gene inactivation in the initiation of EOC. Mice were homozygous for the mutation and crossbred to generate *p53*^*floxP*/*floxP*^*Rb1*^*floxP*/*floxP*^. To assess the efficiency of Cre recombinase (Cre) expression derived by the cytomegalovirus (CMV) promoter, the ovaries were removed and cultured prior to exposure to adenovirus infection. Adenoviruses carrying *CMV-enhanced fluorescent green protein *(Ad*CMVEGFP*) were used as a control against adenoviruses carrying *CMV-Cre *(Ad*CMVCre*). Administration of Ad*CMVCre *resulted in increased cell proliferation assessed by BrdU incorporation. To detect the feasibility in targeting the ovarian bursal cavity in the mouse, Ad*CMVEGFP *was administered. It was detected only in the OSE for 21 days, as expected with a transient adenovirus infection. As a result of both p53 and Rb inactivation, 33 of 34 mice succumbed to ovarian tumors at a median of 227 days. However, administration of an adenovirus to achieve the desired results is cumbersome without generating a reproductive line that would spontaneously form tumors. Targeting the ovarian bursal cavity is difficult at best, making this model not a feasible choice for large-scale applications.

While the previous models developed tumors resembling human serous adenomas, Dinulescu et al. [32] generated mice that have a transcriptionally silent oncogenic allele of *K-ras *(*LSL-K-ras*^*G*12*D*/+^) as first developed by Tyler Jacks [33-35], which can be conditionally expressed through administration of an adenovirus containing Cre. While the *LSL-K-ras*^*G*12*D*/+ ^mice formed benign endometrosis-like lesions and benign lesion within the OSE upon *K-ras *activation, the mice did not form ovarian carcinomas. However, when the *LSL-K-ras*^*G*12*D*/+ ^mice were crossed with *PTEN*^loxP/loxP ^mice, they developed invasive primary ovarian endometrioid adenocarcinomas (OEA), a subtype of EOC, suggesting that phosphate and tensin homologue deletion on chromosome 10 (PTEN) plays a role in tumorigenesis when combined with other oncogenes. This finding is consistent with PTEN deletion or mutation in other cancer types including endometrium, breast, thyroid, intestines, prostate, lung, liver, and T-cell lymphomas [36-40]. Concurrent K-ras and PTEN mutations have also been found in complex endometrial hyperplasias, the precursor type of uterine endometrioid adenocarcinomas [41].

Wu et al. [42] used similar methods to conditionally delete PTEN and adenomatous polyposis coli (APC) tumor suppressor gene upon administration of an adenovirus carrying *Cre*. APC has been shown to regulate Wnt/β-catenin signaling [43]. Wu et al. cross-bred *PTEN*^loxP/loxP ^with *APC*^loxP/loxP ^transgenic mice to determine if there was an interaction between the two pathways. The PTEN^-/-^APC^-/- ^animals developed tumors within 6 weeks upon inactivation, with death occurring at 19 weeks. These tumors resembled human OEA, with increased signaling through Akt. Loss of E-cadherin and cytokeratins indicated that these tumors were undergoing epithelial-mesenchymal transition (EMT), which is consistent with Wnt/β-catenin and PI3K/Akt activation [44,45]. Both the studies by Dinulescu et al. [32] and Wu et al. [42] rely on adenovirus administration and are therefore subject to the same limitations.

Chodanker et al. [46] crossbred mice with follicle stimulating hormone (FSH) receptor promoter fused to Cre recombinase (FSHR-Cre) to mice carrying *Brca1*^loxP/loxP ^to conditionally knockout *Brca1 *in the granulosa cells. Loss of *Brca1 *resulted in multiple cyst formation in 40 of 59 animals (58%) attached to the ovary wall and interior or exterior surface of the uterine horns, which resembled human serous cystademonas, the benign form of ovarian serous carcinomas. One animal formed a solid tumor. Although the FSHR promoter targeted the granulosa cells, the cysts resembled an epithelial morphology as they expressed keratins.

Clark-Knowles et al. [47] used *Brca1*^loxP/loxP ^mice, which upon administration of AdCre would remove introns 5 through 13 (Brca1^Δ55-13^). Conditional deletion of *Brca1 *resulted in morphological changes, such as surface epithelium hyperplasia and formation of inclusion cysts, which was not due to increased proliferation. The incidence of these changes increased over time as observed from 60 days post-infection to 240 days. Interestingly, the genes involved in cancer initiation and progression p53 [48], E-cadherin [49], and Collagen IV [50] were altered in Brca1^Δ55-13 ^ovaries compared to other tumor models. In Brca1^Δ55-13 ^ovaries, p53 was absent compared to SV40-induced tumors. E-cadherin was also downregulated, consistent with preneoplastic transformation. Collagen IV expression was found in the basement membrane, regardless of morphological changes of the OSE.

Building on the report by Connolly et al. [24], El-Naggar et al. [51] used the MISIIR promoter linked to the pituitary tumor-transforming gene (PTTG) to target expression to the OSE. This construct was microinjected into the male pronucleus of CD2F1 embryos to produce transgenic founders. The founders were crossbred with wildtype animals to produce the F1 generation. Positive male and female F1's from the same line were crossbred to produce the F2 generation. While the transgenic females failed to generate any visible tumors, there was an increase in the corpus luteum mass in the transgenic ovaries, which was accompanied by the increase in serum luteinizing hormone (LH) and testosterone levels. The transgenic females also displayed a generalized hypertrophy of the endometrium. This study showed that by using the MISIIR promoter, 3 different tissues could be targeted: OSE, granulosa cells, and pituitary.

More recently, Liang et al. [52] used the MISIIR promoter to drive expression of murine phosphatidylinositol 3-kinase catalytic subunit p110-alpha (PIK3CA) in transgenic mice. Although over-expression of PIK3CA resulted in increased phosphorylated Akt as its downstream target and in OSE hyperplasia, after 18 months post-birth of the F1 generation, tumorigenesis did not occur. Interestingly, the authors cultured isolated ovaries from non-transgenic mice and co-transfected them with both *PIK3CA *and mutant *K-ras *or *c-myc *to assess OSE transformation *in vitro*. Concurrent over-expression of *PIK3CA *and mutant *K-ras *led to increased anchorage-independent growth of cultured OSE cells. Liang et al. [52] acknowledged that producing a "bigenic" animal by crossbreeding the transgenic PIK3CA mouse with a transgenic mutant K-ras remains a technical challenge because mutant K-ras animals develop tumors that inhibit reproduction. However, they suggested that a Cre-lox system of K-ras expression may provide an alternative method of generation.

### Genetically induced granulosa cell tumor (GCT) models

Granulosa cell tumors (GCT) represent 2-5% of all OCs [53] arising from the granulosa cells of the ovary, which are responsible for estradiol production. Therefore, GCT are also called sex cord-stromal tumors. One of the first GCT models was produced by Kananen et al. [54], who fused the inhibin α-subunit promoter to SV40 TAg to generate transgenic founders. Three lines were established from these founders with all transgenic offspring developing GCT in two of the lines: 14/14 animals in one line and 22/22 animals in another. The granulosa cells still maintained their receptors, making them responsive to gonadotrope stimulation. SV40 TAg mRNA expression was found in the gonads, adrenal glands, pituitary, and brain indicating leakiness of the inhibin α-subunit promoter.

Nilson et al. [55] generated a GCT tumor model through chronic hyperstimulation of LH by fusing the β-subunit of LH containing a carboxy-terminal peptide of human chorionic gonadotropin β subunit to a bovine inhibin α-subunit promoter (α-LHβCTP) to extend its half-life and target gonadotrope cells. As a result of the constant LH stimulation, the ovary became anovulatory from its inability to respond to the necessary LH surge. While the animals could be super ovulated, the pregnancy failed at mid-gestation. Females also displayed a reduction in the amount of primordial follicles with an increase in large hemorrhagic follicles. By 5 months of age, the females developed GCT and pituitary hyperplasia, dying shortly thereafter due to bladder atony and kidney failure.

Selvakumaran et al. [56] isolated a new promoter to determine specificity to the ovary by using repetitive retrovirus-like elements in the rat genome, termed ovarian-specific transcription units (OSTUs). The U3 region of the OSTUs was cloned and renamed ovarian-specific promoter-1 (OSP-1). OSP-1 was then used by Garson et al. [43] to drive expression of SV40 TAg (OSP-TAg). While successfully producing both male and female founders, many females either failed to reproduce or the offspring failed to develop tumors despite high levels of expression of TAg. Two of the three female founders developed GCT, but expression was not restricted to the ovary as osteosarcomas formed in the liver and lung. The thymus also showed enlargement demonstrating that OSP-1 was sufficiently leaky. Male founders also expressed TAg in a variety of tissues including testes, liver, and lung, but failed to produce any tumors.

Boerboom et al. [57] showed that constitutive activation of β-catenin in granulosa cells of transgenic mice (*Catnb*^flox(ex3)^*Amhr2*^cre/+^) produced GCT. *Cre *knocked into the *anti-Mullerian hormone receptor, type II (AMHR2) *gene, designated *AMHR2*^cre/*cre*^, to localize its expression. Exon 3 of β-catenin encodes for multiple phosphorylation sites that are necessary for its degradation, while its removal maintains the protein's functionality. However, the excision of exon 3 of *Catnb *by Cre was a relatively inefficient process as few *Catnb*^flox(ex3)^*Amhr2*^cre/+ ^mice displayed abnormal expression of β-catenin. Histochemical analysis showed that the ovaries of 3 to 24-week-old transgenic mice developed abnormal follicle-like structures consisting of pleiomorphic granulosa cells without the presence of an oocyte, resulting in sub-fertility due to an impaired follicular response that could be overcome with age at the end of the third month. GCT were seen at 19 weeks with the incidence of formation over time to 57% at 7.5 months.

Building upon the previous study, Lague et al. [58] conditionally deleted PTEN in the granulosa cells by crossbreeding *PTEN*^flox/flox^with *AMHR2*^cre/*cre *^mice to create *PTEN*^flox/flox^*AMHR*^cre/+^. Most *PTEN*^flox/flox^*AMHR*^cre/+ ^mice failed to generate any ovarian abnormalities; while these animals could establish pregnancies, they failed to carry the litter to term or had small litters due to fetal death. However, 5 of 70 (~ 7%) female *PTEN*^flox/flox^*AMHR*^cre/+ ^developed ovarian tumors. Four of the 5 were bilateral tumors developing between 7 weeks and 7 months that were identified as GCT. *PTEN*^flox/flox^*AMHR*^cre/+ ^mice also developed tumor cell emboli and metastases in the lungs. *PTEN*^flox/flox^*AMHR*^cre/+ ^GCT showed altered PI3K/Akt signaling, with increases in both phosphorylated Akt and mammalian target of rapamycin (mTOR) levels compared to normal granulosa cells. Furthermore, to determine if the PI3K/Akt pathway could cross-talk with the WNT/CTNNB1 (encoding β-catenin) pathway, they constitutively activated both pathways using the mouse model *PTEN*^flox/flox^*CTNNB1*^flox(ex3)^*AMHR*^cre/+^. These mice developed bilaterial ovarian tumors with 100% penetrance at an early age. Dysplastic cells were seen in the ovaries of newborn mice and 20.5-day embryos suggesting that this occurs perinatally. The ovarian tumors visibly distended the abdomen by 5 weeks of age with death occurring before 9 weeks, possibly due to severe anemia. Pulmonary emboli were also seen in *PTEN*^flox/flox^*CTNNB1*^flox(ex3)^*AMHR*^cre/+ ^mice.

## Conclusion

The syngeneic model has shown that MOSE cells are capable of spontaneously transforming into a tumorigenic phenotype with repeated passages, indicating that repeated repair of the OSE as a result of excessive ovulation could be a cause of tumorigenesis. The manipulation of MOSE cells and subsequent injection may form a tumor, but the tumor could form solely from the MOSE cells and not the host OSE cells as MOSE cells could undergo mesenchymal-epithelial transition (MET) to imbed in the host tissue. The limitation of extracting MOSE cells and culturing them before transplantation allows for only a limited number of animals to be produced and does not establish an inheritable line that would spontaneously form EOC.

A summary of the genetically induced ovarian epithelial tumor models can be found in Table 1. These models have provided valuable information regarding gene dysfunction necessary for tumorigenesis, including p53 and Rb deletion, as well as over-expression of known oncogenes *c-myc, Kras*, and *Akt*. Models that use transgene expression during embryonic development do not accurately represent spontaneous EOC, which tends to occur in post-menopausal women, and yet gene deletion by adenoviruses carrying *Cre *allows for only transient expression. Some of these models have been successful in producing ovarian tumors; however, the aggressiveness of tumor formation can inhibit reproduction and limit establishment of a reproductive line. These models are limited by the lack of a specific promoter for the ovaries, as the MISIIR and keratin 5 promoter are both leaky. Clearly, the need to produce a model that can recapitulate human EOC is still necessary to understand the etiology of a very complex disease to allow for better screening and treatment purposes.

**Table 1:** Summary of promoters and targeted genes for ovarian epithelial tumorigenesis.

**Authors**	**Promoter**	**Targeted gene**	**Tumorigenesis**	**Limitation**
Orsulic et al. (2002)	keratin-5, RCAS	TVA, p53^-/-^, oncogenes	Yes	External manipulation
Connolly et al. (2003)	MISIIR	SV40 TAg	Yes	Inhibited female reproduction
Flesken-Nikkita et al. (2003)	AdCre	p53^-/- ^& Rb^-/-^	Yes	Transient expression
Dinulescu et al. (2005)	AdCre	K-ras & PTEN^-/-^	Yes	Transient expression
Wu et al. (2007)	AdCre	PTEN^-/- ^& APC^-/-^	Yes	Transient expression
Chondankar et al. (2005)	FSHR	Cre, BRCA1^-/-^	No	
Clark-Knowles et al. (2007)	AdCre	BRCA1^Δ5-13^	No	Transient expression
El-Naggar et al. (2007)	MISIIR	PTTG	No	
Liang et al. (2009)	MISIIR	PIK3CA	No	

Table 2 summarizes the genetically-induced GCT models. OSP-1 and inhibin α-subunit promoters are not specific to the ovaries, although sufficiently strong to drive tumorigenesis. While knocking *Cre *into the *AMHR2 *locus was a clever design, the efficiency of the targeted gene deletion was relatively ineffective, as gene expression was maintained, possibly due to Cre acting on only the cis chromosome so ovarian abnormalities were not observed.

**Table 2:** Summary of promoter and targeted genes of granulosa cell tumors (GCT).

**Authors**	**Promoter**	**Targeted gene**	**Tumorigenesis**	**Limitation**
Kananen et al. (1995)	α-subunit of inhibin	SV40 TAg	Yes	
Nilson et al. (2000)	α-subunit of inhibin	LHβCTP	Yes	Females unable to reproduce
Garson et al. (2003)	OSP-1	SV40 TAg	Yes	developed tumors
Boerboom et al. (2005)	MISIIR/Cre	mutant β-catenin	Yes	Transient expression
Lague et al. (2008)	MISIIR/Cre	PTEN^-/- ^& CTNNB1^-/-^	Yes	Transient expression

Many models have used *SV40 TAg*, a monkey virus belonging to the polyomavirus family, to initiate tumorigenesis. In a breast cancer model, SV40 TAg was shown to inactivate p53 and Rb to initiate tumorigenesis [59]. While SV40 TAg has been reported in several types of human cancer including breast, brain, osteocarcomas, lymphomas, hepatocellular carcinomas, papillary thyroid carcinomas, and pleural mesothelioma [60-66], it has not been reported in OC. At best, SV40 TAg has been used widely to immortalize OC cell lines [67-69]. Moreover, SV40 TAg immortalization of cultured human OSE cells eliminated the presence of CA-125 [69], one of the current diagnostic markers for EOC [70].

To understand the complexity of OC, a mouse model representing each subtype is needed. From the current transgenic models, we have learned that different pathways are used for tumorigenesis. For EOC, p53 mutations/inactivation plays a role, as seen in high-grade tumors [26], while GCT have intact p53 but dysregulated PTEN and Wnt/β-catenin signaling occurring perinatally [42,57,58].

## List of Abbreviations

α-LHβCTP: inhibin α-subunit promoter, *LH *gene with a carboxy-terminal peptide of human chorionic gonadotropin β subunit attached; Ad*CMVCre*: adenoviruses containing CMV promoter and *Cre *gene; Ad*CMVEGFP*: adenoviruses containing CMV promoter and *EGFP *gene; *AMHR2: anti-Mullerian hormone receptor, type II; *APC: adenomatous polyposis coli; *Catnb*^flox(ex3)^*Amhr2*^cre/+^: transgenic mice that Cre knocked into the *AMHR2 *gene to produce constitutive activation of β-catenin; CMV: cytomegalovirus; Cre: Cre recombinase; DMBA: 7,12-Dimethylbenz(a)anthracene; EMT: epithelial-mesenchymal transition; EOC: epithelial ovarian cancer; FSHR: follicle stimulating hormone receptor; GCT: Granulosa cell tumors; LH: luteinizing hormone; *LSL-K-ras*^*G*12*D*/+^: mice that have a transcriptionally silent, oncogenic allele of *K-ras*; MET: mesenchymal-epithelial transition; MISIIR: Mullerian Inhibitory Substance Type II Receptor; MOSE: mouse ovarian surface epithelium; OEA: ovarian endometrioid adenocarcinomas; OSE: ovarian surface epithelium; OSP-1: ovarian-specific promoter-1; OSTUs: ovarian-specific transcription units; PIK3CA: catalytic subunit p110-alpha of phosphatidylinositol 3-kinase; PTEN: phosphate and tensin homologue deleted on chromosome 10; PTTG: pituitary tumor-transforming gene; RCAS: replication-competent avian leukosis viral-derived vectors; SV40 TAg: simian virus 40 T antigen; TVA/p53^-/-^: transgenic mice expressing TVA receptor and are null for p53.

## Competing interests

The authors declare that they have no competing interests.

## Authors' contributions

MYF drafted the manuscript. SSK participated in substantial contribution to conception and revising of the manuscript. All authors read and approved the final manuscript.
